# Treatment of Chronic Neck Pain with Transcranial Direct Current Stimulation: A Single-Blinded Randomized Clinical Trial

**DOI:** 10.3390/biomedicines13071746

**Published:** 2025-07-17

**Authors:** Manuel Rodríguez-Huguet, Miguel Ángel Rosety-Rodríguez, Daniel Rodríguez-Almagro, Rocío Martín-Valero, Maria Jesus Vinolo-Gil, Jorge Bastos-Garcia, Jorge Góngora-Rodríguez

**Affiliations:** 1 Department of Nursing and Physiotherapy, University of Cádiz, 11009 Cádiz, Spain; manuel.rodriguez@uca.es (M.R.-H.); mariajesus.vinolo@uca.es (M.J.V.-G.); jorge.gongora@uca.es (J.G.-R.); 2 MOVE-IT Research Group, Department of Physical Education, Faculty of Education Sciences, University of Cádiz, 11519 Puerto Real, Spain; 3 Biomedical Research and Innovation Institute of Cádiz (INiBICA), Puerta del Mar University Hospital, University of Cádiz, 11009 Cádiz, Spain; 4 Department of Nursing, Physiotherapy and Medicine, University of Almería, 04120 Almería, Spain; dra243@ual.es; 5 Department of Physiotherapy, Faculty of Health Sciences, University of Málaga, 29071 Málaga, Spain; rovalemas@uma.es; 6 Rehabilitation Clinical Management Unit, Interlevels-Intercenters Hospital Puerta del Mar, Hospital Puerto Real, Cádiz Bay-La Janda Health District, 11009 Cádiz, Spain; 7 Independent Researcher, 11003 Cádiz, Spain; jorgen.bastosgarcia@gmail.com

**Keywords:** chronic pain, neck pain, physical therapy, transcranial direct current stimulation, transcutaneous electric nerve stimulation

## Abstract

**Background/Objectives**: Neck pain is defined as an unpleasant sensory and emotional experience associated with actual or potential tissue damage, affecting the cervical region. It represents one of the leading causes of disability, with a prevalence of 30%. Transcranial direct current stimulation (tDCS) is a non-invasive electrotherapy technique that enables direct modulation of cortical excitability. It involves the application of a low-intensity electrical current to the scalp, targeting the central nervous system. The aim of this study was to analyze the effects of tDCS on functionality, pain, mobility, and pressure pain threshold in patients with chronic nonspecific neck pain. **Methods**: Thirty participants (18–60 years) were selected to receive ten treatment sessions over a four-week period using tDCS (CG = 15) or transcutaneous electrical nerve stimulation (TENS) (CG = 15), with the following various related variables evaluated: functionality (Neck Disability Index), pain intensity (NPRS), cervical range of motion (ROM), and pressure pain threshold (PPT). Assessments were conducted at baseline, post-treatment, one month, and three months after the intervention. **Results**: The within-group analysis revealed statistically significant improvements for both groups at post-treatment, one-month follow-up, and three-month follow-up. **Conclusions**: The comparison between groups shows favorable changes in the tDCS group for PPT measurements.

## 1. Introduction

Neck pain, cervical pain, or cervicalgia is defined as an unpleasant sensory and emotional experience. It is associated with actual or potential tissue damage affecting the cervical region, which extends from the suboccipital line to the level of the scapular spine [1]. Cervical pain is among the leading causes of disability, with a prevalence exceeding 30% [1,2,3] and a substantial socioeconomic impact [1,2,3,4]. In about half of the cases it becomes chronic, with recurrent pain episodes that may last beyond six months [2,5].

Neck pain is typically nonspecific, meaning it cannot be attributed to fractures, trauma, or identifiable pathological conditions. Therefore, clinical assessment must focus on ruling out red flags suggestive of a specific systemic origin [1,2,5,6]. Currently, it is crucial to explore new therapeutic approaches for chronic pain management, particularly due to the limited efficacy of available pharmacological treatments. It is essential to identify alternative options that are effective and well-tolerated by patients [6,7].

The assessments of patients with cervicalgia involve evaluating pain intensity, using the Numerical Pain Rating Scale (NPRS), disability or functional impairment, using Neck Disability Index (NDI), and the range of motion of the cervical region [5,8]. In addition, it is essential to identify comorbidities and associated symptoms [9].

Transcranial direct current stimulation (tDCS) is a non-invasive, painless electrotherapy technique aimed at treating the central nervous system, particularly chronic pain [10,11,12,13], improving functionality and reducing symptoms [14]. This therapy involves the application of a low-amplitude, monophasic electrical current directly to the skull, using two electrodes integrated into a helmet placed on the scalp [7,11]. This technique allows for the direct modulation of the excitability of specific cortical areas [15], inducing alterations in cortical excitability to normalize it due to changes in the resting membrane potential [16,17,18,19,20,21]. Researchers suggest that tDCS can reduce pain intensity [7,12,13,15,16,18,22], reporting sustained effects lasting up to 12 weeks [19].

It is estimated that a minimum duration of three minutes and an intensity of at least 0.4 mA are required during tDCS applications to induce changes in cortical excitability that persist beyond the stimulation period [12]. However, the intensity ranges from 1 to 2 mA, with an accepted application time of around 20 min [7,11,12,15,18,19,20,21,22,23,24,25].

Adverse effects of tDCS are minimal and considered negligible [11,12,18]. Patients typically report a mild tingling sensation on the scalp, which subsides shortly after the stimulation begins, and skin lesions are not reported [16,17]. Furthermore, depending on the condition being treated, tDCS can be combined with therapeutic exercise [26].

Transcutaneous electrical nerve stimulation (TENS) has been shown to be effective for neuropathic, nociceptive, and musculoskeletal pain. Its analgesic effect is generally attributed due to the blockade of nociceptive signal transmission, specifically, at the peripheral level, the electrical current stimulates nerve fibers (A–β or A–δ), which decreases nociceptor activity and modulates ion channels in the nerves, thereby curbing pain transmission [27]. This technique is simple and cost-effective. Evidence suggests that TENS treatment should be applied in continuous mode with frequencies ranging from 60 to 100 Hz, pulse widths between 40 and 250 microseconds, and an intensity tolerable to the patient [28].

Other treatments have been shown to be effective in reducing neck pain. The application of therapeutic ultrasound may reduce pain intensity; however, it is not yet established whether its combination with conventional treatments provides additional benefits [29]. Exercise-based interventions are currently the best evidence-based treatment for managing chronic cervical pain [30]. Physical exercise interventions have been demonstrated to be effective in reducing pain intensity and lowering the risk of a future episode withing the following 12 months. Nevertheless, at present, there is no consensus regarding the optimal structure of an exercise program, as it should be personalized to address the specific requirements and needs of each individual [30,31]. Physical therapy is crucial for restoring movement and reducing pain. Therefore, it is essential to design and implement highly effective clinical treatments for neck pain.

The aim of this study was to analyze the effects of tDCS on functionality, pain, mobility, and pressure pain threshold in patients with chronic nonspecific cervical pain and conduct a comparison between tDCS and TENS.

It was hypothesized that the application of tDCS will produce superior benefits compared to conventional treatment with TENS allowing for a direct comparison between two replicable electrotherapy treatment methods with clearly defined application parameters.

Therefore, the present study opens a new avenue of research, offering a deeper understanding of the treatment of chronic neck pain through specific physical therapy interventions.

## 2. Materials and Methods

### 2.1. Study Design

The design of the present study was an experimental, longitudinal, quantitative, single-blind randomized clinical trial using medical instruments. The blinding of this study was inherently challenged; physiotherapists could not be blinded, and participants were likely able to infer their assigned group due to the distinct sensory perceptions associated with each treatment. The study was identified at ClinicalTrials.gov: NCT04729270 on 6 February 2023. It was performed in accordance with the Helsinki Declaration and the current legal regulations, accepted by research Cádiz Research Ethics Committee (registration number: 169.22; date: 2 February 2023).

### 2.2. Participants and Recruitment

The considered criteria included adults, men or women, with an age range between 18 and 60 years. All participants had a diagnosis of nonspecific chronic cervicalgia, characterized by pain persisting for more than three months and without a systemic origin [32,33]. Their condition was evaluated and diagnosed by a traumatology and orthopedic specialist using assessment scales outlined in clinical practice guidelines. This examination included an assessment of the Numerical Pain Rating Scale (NPRS) and Neck Disability Index (NDI). The presence of red flags was ruled out, and supplementary imaging tests were requested at the discretion of the physician [32,34]. Individuals were excluded if they were previously treated with tDCS, had undergone brain surgery or had metallic implants in the skull, presented with neurological disorders or alcohol/drug dependence, had experienced trauma within one month prior, had fibromyalgia, pacemakers, were pregnant, or were undergoing contraceptive treatment.

Participants were recruited from Policlínica Santa María Clinic. The random sequence for allocation to treatment groups was generated by an independent researcher and was concealed in sequentially numbered envelopes. The randomized sequence was designed to ensure balanced groups based on the number of participants assigned. The sample size was calculated using a random allocation software program (Epidat 4.2) (Servicio de Epidemiología de la Dirección Xeral de Saúde Pública da Consellería de Sanidade, Xunta de Galicia, Santiago de Compostela, Spain) based on data by Lauche et al. [35] to detect statistically significant differences in pain intensity using NPRS, with 80% statistical power and 95% confidence level. Therefore, it was determined that a minimum sample size of 15 participants per group was required for conducting a study of this nature. Data collection was conducted by a physician who was blinded as to which participants received tDCS or TENS intervention. A total of 30 participants was included in the present study, with 60% of the sample being female, and a mean age of 36,7 years (SD = 9,9); 15 participants were assigned to the intervention group (IG = 15) and 15 to the control group (CG = 15). Table 1 displays the baseline morphological and clinical characteristics of the sample. The baseline comparability analysis revealed no statistically significant differences between the intervention and control groups in most variables. The only exception was cervical extension, which showed a significant difference between groups (*p* = 0.004).

### 2.3. Measurements

Study variables were measured at baseline, immediately after the intervention, and at one and three months before the intervention. The measurements data were always collected by a collaborating physiotherapist, who was blinded as the evaluator.

Pain intensity, assessed using NPRS [36], was designated as the primary outcome variable. Functionality (assessed using the NDI [8]), pressure pain, and range of motion were designated as secondary variables. Pressure pain was assessed using a pressure algometer (Wagner, Baseline FPK, Greenwich, CT, USA), a device used to measure the pressure pain threshold (PPT) at a specific point by applying the minimum force required to induce pain. Evaluations were conducted bilaterally over the upper trapezius and splenius muscles. It is a validated and reliable method, with a high correlation coefficient (>0.91) [37,38,39,40,41]. Cervical range of motion (ROM) was measured with a digital inclinometer (Baseline, India). The inclinometer is reliable, ensuring that any observed variation is not attributed to measurement errors or natural fluctuations between trials [42].

### 2.4. Procedures

**Figure 1:**
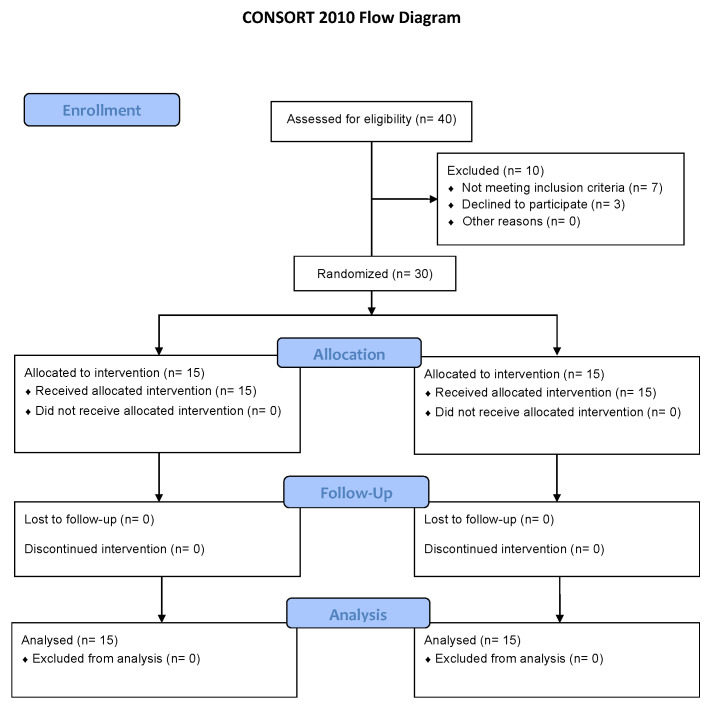
CONSORT flow diagram.

The development of the intervention in both groups was the same. All participants performed ten treatment sessions distributed across four weeks (Figure 1). The intervention group (tDCS group) received transcranial direct current stimulation (tDCS), while the control group (TENS group) was treated with transcutaneous electrical nerve stimulation (TENS).

Patients of the tDCS group received 20 min of stimulation at an intensity of 2 mA. Electrode 35 cm^2^ placement was configured to deliver anodal stimulation over the primary motor cortex (M1), the cathode was positioned over the orbitofrontal cortex (OF) to complete the circuit [24,25], and the anode was placed on the hemisphere opposite the side of predominant pain. Participants of the TENS group also received the mentioned 10 sessions, with a current application time of 20 min to match the treatment duration of the experimental group. Electrodes were placed bilaterally over the cervical region, specifically, self-adhesive electrodes were used on the upper trapezius and suboccipital muscles, with a frequency of 100 Hz, pulse duration of 250 µs, and an intensity adjusted to a tolerable level [28].

### 2.5. Statistical Analysis

The data were analyzed using IBM SPSS Statistics for Mac (e.g., 29.0.1.1; IBM Corp., Armonk, NY, USA) [43]. Statistical significance was established at a threshold of *p* < 0.05. Descriptive analyses were conducted, with continuous variables expressed as means and standard deviations, and categorical variables as frequencies and percentages. To assess assumptions of normality and homogeneity of variances, the Kolmogorov–Smirnov test and Levene’s test were employed, respectively. Baseline comparability between groups was examined using Student’s t-test for continuous variables and the Chi-square test for categorical variables.

To evaluate group-by-time effects, a 2 × 4 mixed-model repeated measures analysis of variance (ANOVA) was performed, with particular focus on the interaction between time and group. When initial differences between groups were detected, treatment effects were assessed using analysis of covariance (ANCOVA), controlling for any baseline group differences by including the relevant baseline variable as a covariate. Between-group comparisons at post-treatment, one-month follow-up, and three-month follow-up were conducted using Student’s t-tests on the change scores from baseline. Within-group differences across these time points relative to baseline were assessed using paired samples t-tests. Bonferroni correction was applied to adjust for multiple comparisons, setting the significance level at 0.017 for between-group analyses and 0.0125 for within-group analyses.

The effect size (ES) for the time-by-group interaction in the 2 × 4 mixed ANOVA was assessed using partial eta-squared (ηp^2^). This statistic represents the proportion of variance in the dependent variable that is attributable to a specific effect, after accounting for other sources of variance, including error. It is calculated by dividing the sum of squares for the effect by the sum of the effect sum of squares and its associated error term. According to Cohen, eta-squared can be deemed insignificant when <0.02, small if between 0.02 and 0.15, medium if between 0.15 and 0.35, and large if >0.35 [44].

For bivariate analyses, Cohen’s d was used to quantify the ES. It was computed by dividing the mean difference between the two groups by the pooled standard deviation. Cohen’s d can be interpreted using conventional thresholds: values below 0.2 indicate a negligible effect; values between 0.2 and 0.5 suggest a small effect; values between 0.5 and 0.8 denote a moderate effect; and values greater than 0.8 are considered indicative of a large effect [43].

## 3. Results

The repeated measures ANOVA conducted to assess time-by-group interaction showed statistically significant differences for cervical extension (F = 3.316, *p* = 0.036, ηp^2^ = 0.285), right cervical rotation (F = 6.751, *p* = 0.002, ηp^2^ = 0.438), and PPT at all assessed variables (Table 2). Specifically, significant interactions were found for the right trapezius (F = 6.052, *p* = 0.003, ηp^2^ = 0.411), left trapezius (F = 5.338, *p* = 0.005, ηp^2^ = 0.381), right splenius (F = 3.132, *p* = 0.043, ηp^2^ = 0.265), and left splenius (F = 3.606, *p* = 0.027, ηp^2^ = 0.294).

The findings indicate that the evolution of neck mobility and pain sensitivity was distinct and significant between the groups, with medium to large effect sizes. This suggests that the proposed therapies offer benefits that warrant in-depth study.

The within-group analysis revealed statistically significant improvements in all outcome variables for both groups at post-treatment, one-month follow-up, and three-month follow-up, except for the PPT of the right trapezius in the control group at post-treatment, which did not reach statistical significance (MD = 0.8; *p* = 0.019) (Table 3).

The between-group analysis revealed statistically significant improvements of greater magnitude in the intervention group compared to the control group in all parameters related to pressure pain threshold (PPT), as shown in Table 3: right trapezius PPT (MD = 0.5; *p* = 0.010; d = 1.015), left trapezius PPT (MD = 0.4; *p* = 0.005; d = 1.125), right splenius PPT (MD = 0.3; *p* = 0.010; d = 1.006), and left splenius PPT (MD = 0.3; *p* = 0.017; d = 0.927). Additionally, a significantly greater improvement was also observed in the intervention group compared to the control group in right cervical rotation (MD = 7.1; *p* = 0.007; d = 1.062) (Table 3).

## 4. Discussion

As previously mentioned, chronic cervical pain is one of the most frequent reasons for physiotherapy consultation. It is typically a recurrent condition that significantly limits functional capacity and independence in daily life. Our pre-intervention assessment of the study population consistently showed this. The evaluation of specific electrotherapy-based treatment methods could help establish effective and replicable treatments that are of interest for clinical practice. Thus, the present study was conducted under the hypothesis that tDCS could improve symptomatology in patients with chronic cervical pain, proving a more effective therapy than conventional TENS electrotherapy.

Overall, the results were positive. Both groups showed improvements from baseline across all outcome measures (NPRS, NDI, ROM, and PPT) at both post-treatment and long-term follow-up assessments. However, direct comparison between the experimental tDCS group and the conventional treatment group showed minimal differences. This suggests that while tDCS might be a viable treatment option for chronic cervical pain patients, it does not appear to significantly outperform standard electrotherapy interventions like TENS. Therefore, these techniques warrant consideration as part of a multimodal treatment approach that must be individualized to meet each patient’s specific needs. Future research should consider longer follow-up periods to observe potential long-term differences, alongside developing screening strategies or retrospective studies to analyze the pre-existing duration of the condition.

The present study evaluates the effects of anodal tDCS applied over the primary motor cortex M1 on resting-state brain signals to improve for the treatment of chronic nonspecific cervical pain, in comparison with standard therapy using TENS. M1 stimulation has demonstrated beneficial effects on pain reduction, primarily through its influence modulation of nociceptive signaling pathways [24,45,46]. Furthermore, as M1 stimulation has been associated with modulation of the μ-opioid system, it represents a strategically favorable target (M1-tDCS) in the management of pain [46,47,48].

The precise placement of each electrode is crucial to achieving the desired effects, as it determines the current delivered to the central nervous system [17]. Research also examines the separate effects of anodal over the M1 and left dorsolateral cortex (F3) regions on pain relief in patients with type 2 diabetes suffering from neuropathic pain. Results showed that pain intensity was significantly lower in both groups. However, M1 stimulation demonstrated superior outcomes compared to F3 [49]. As shown in recent studies, other techniques of non-invasive neuromodulation, such as low-intensity focused ultrasound pulsation, suggest that the selection of stimulation modality and target area could influence treatment outcomes depending on the neurophysiological of the patient, potentially surpassing the effects observed through tDCS or TENS, even with a lower number of sessions [50]. Our findings are consistent with recent burgeoning research demonstrating the capacity of tDCS to elicit substantial improvements in upper limb functionality. This corroborates the evolving evidence base supporting tDCS as a valuable rehabilitative modality, thereby expanding therapeutic avenues for conditions impacting the mobility and the intricate neuro-mechanical relationship between the cervical spine and upper extremity [51]. Consequently, the application of tDCS could support the anodic placement of electrodes over the hemisphere opposite the dominant or painful side. Further comparative research is needed in this area.

Considering the results and existing evidence, it may be necessary to re-evaluate current dosages, application modalities, and even treatment frequency, always prioritizing patient safety. Another crucial aspect to explore is whether the observed effects are directly attributable to the treatments or if they might be associated with the natural regression of the pathology. When comparing tDCS and TENS, it is important to note their distinct mechanisms: TENS directly stimulates afferent nerve pathways in the perceived painful area, whereas tDCS involves direct application to the central nervous system. Both approaches could be equally valid. Furthermore, the nearly imperceptible nature of tDCS could offer a significant advantage for patient comfort.

Our findings, in line with the recent literature, demonstrate that tDCS is effective in reducing continuous and paroxysmal neuropathic pain [7,12,13,15,16,21,25,52]. Moreover, tDCS stimulation in M1 in brachial plexus injury patients also promoted improvements in anxiety-state, but not in quality of life [53].

The analgesic effects of TENS are commonly attributed to the blockade of nociceptive signal transmission [27]. The modest reduction in pain observed in the control group, treated with TENS, may be attributed to a placebo effect, as suggested by several researchers [54]. Given the extensive use of TENS currents, it would be beneficial to further elucidate their effects, particularly since their application frequently lacks proper supervision and tailored adjustment to individual patient or disorder requirements.

The primary limitations of this study were the inherent challenges in blinding both patients and physiotherapists. Additionally, the isolated application of passive techniques allows for a clear evaluation of each technique’s specific effects without interference (a significant strength of this research). The sample size of the investigation could be considered another limitation in our study, which makes it difficult to extrapolate the results to the global population. However, we believe that the results of this study provide strong preliminary observation. Future studies should explore combining treatments like tDCS with active exercise modalities and patient education to promote movement, adopting a biopsychosocial approach.

Future research should explore and evaluate different application parameters, including intensity, duration, and polarity. Additionally, the potential influence of the placebo effect and patient perceptions on reported treatment outcomes should be acknowledged [12,18,55]. It is essential to accurately determine the therapeutic benefits and establish optimal dosing parameters [14]. Furthermore, the feasibility of applying these treatments in home-based settings warrants further investigation [7,18,53], as does exploring the individualization of stimulation protocols tailored to each patient’s specific needs [7,19].

## 5. Conclusions

In patients with chronic neck pain, both tDCS and conventional TENS therapies elicited beneficial outcomes across multiple parameters, including pain (NPRS), functional capacity (NDI), range of motion (ROM), and pressure pain threshold (PPT). While tDCS showed comparable efficacy to TENS and even some favorable changes in PPT measurements, it did not significantly outperform it overall. Given its gentle application, tDCS is a promising treatment option. Future research should focus on optimizing tDCS parameters, exploring its integration into multimodal biopsychosocial interventions with active exercise and patient education, and exploring its use for home-based care.

## Figures and Tables

**Table 1:** Morphological and clinical characteristics of sample.

	ALL (30)	IG (15)	CG (15)	*p*
Categorical	Frequency (%)	Frequency (%)	Frequency (%)	
Sex	Male	12 (40.0)	4 (26.7)	8 (53.3)
	Female	18 (60.0)	11 (73.3)	7 (46.7)
Continuous	Mean (SD)	Mean (SD)	Mean (SD)	*p*
Age (years)	36.7 (9.9)	31.6 (7.0)	40.7(10.5)	0.854
Body Mass (kg)	64.8 (13.4)	59.2 (10.1)	70.5 (14.3)	0.507
Height (m)	1.7 (0.1)	1.6 (0.1)	1.7 (0.04)	0.520
BMI (kg/m^2^)	23.4 (4.4)	22.5 (4.0)	24.4 (4.8)	0.138
NPRS	6.8 (0.8)	6.8 (0.9)	6.8 (0.7)	1.000
NDI	25.7 (6.6)	24.9 (7.0)	26.4 (6.2)	0.551
Range of Motion (degrees)				
Flexion	27.1 (4.0)	26.6 (4.1)	27.5 (4.0)	0.534
Extension	31.7 (5.7)	34.5 (5.5)	28.9 (4.3)	0.004
Right Side Bending	39.2 (4.2)	39.8 (4.5)	38.5 (4.0)	0.421
Left Side Bending	38.2 (3.6)	38.9 (3.8)	37.6 (3.4)	0.345
Right Rotation	43.7 (6.6)	42.4 (6.1)	45.1 (7.0)	0.273
Left Rotation	53.5 (6.0)	53.4 (5.6)	53.5 (6.7)	0.953
Pressure Pain Threshold (kg/cm^2^)				
Right Trapezius	1.9 (0.5)	1.9 (0.6)	1.9 (0.4)	0.725
Left Trapezius	1.6 (0.5)	1.9 (0.5)	1.8 (0.5)	0.608
Right Splenius	1.5 (0.4)	1.5 (0.5)	1.4 (0.3)	0.276
Left Splenius	1.5 (0.4)	1.6 (0.5)	1.4 (0.3)	0.309

Abbreviations. BMI: Body Mass Index; SD: Standard Deviation; NPRS: Numerical Pain Rating Scale; NDI: Neck Disability Index.

**Table 2:** Statistical significance, effect size, and statistical power of time-by-group interaction from analysis of variance.

Variable	F	*p*	ηp^2^	Power
NPRS	0.716	0.551	0.076	0.181
NDI	1.239	0.316	0.125	0.292
Range of Motion (degrees)				
Flexion	0.824	0.493	0.087	0.203
Extension	3.316	0.036	0.285	0.684
Right Side Bending	1.044	0.389	0.108	0.250
Left Side Bending	0.009	0.999	0.001	0.051
Right Rotation	6.751	0.002	0.438	0.952
Left Rotation	1.680	0.196	0.162	0.387
Pressure Pain Threshold (kg/cm^2^)				
Right Trapezius	6.052	0.003	0.411	0.927
Left Trapezius	5.338	0.005	0.381	0.890
Right Splenius	3.132	0.043	0.265	0.659
Left Splenius	3.606	0.027	0.294	0.727

Abbreviations. BMI: Body Mass Index; SD: Standard Deviation; NPRS: Numerical Pain Rating Scale; NDI: Neck Disability Index. *p*-value set at 0.05.

**Table 3:** Between-group differences and within-group change scores in instability at one week, one month, and three months.

Variables		T1	T2	T3	Differences T1 to T2				Differences T1 to T3				Differences T1 to T3			
					Within-Group Changes	Mean Between-Group Differences			Within-Group Changes	Mean Between-Group Differences			Within-Group Changes	Mean Between-Group Differences		
		Mean (SD)				MD	*p*	*d*	MD (SD)	MD		*p*	MD (SD)	MD	*p*	*d*
NPRS	IG	2.9 (0.8)	1.9 (1.2)	1.6 (1.2)	−3.9 (0.9) **	−0.4	0.359	−0.340	−4.9 (1.4) **	−0.7	0.142	−0.552	−5.2 (1.4) **	−0.7	0.174	−0.509
	CG	3.3 (1.1)	2.7 (1.2)	2.3 (1.3)	−3.5 (1.4) **				−4.1 (1.3) **				−4.5 (1.5) **			
NDI	IG	13.9 (4.2)	9.5 (5.1)	8.2 (5.7)	−11.0 (4.3) **	0.7	0.684	0.150	−15.4 (5.2) **	−0.7	0.663	−0.161	−16.7 (6.1) **	−0.3	0.892	−0.050
	CG	14.7 (4.9)	11.7 (4.6)	9.9 (4.0)	−11.7 (4.6) **				−14.7 (3.8) **				−16.5 (4.5) **			
Range of Motion		(degrees)														
Flexion	IG	31.8 (3.5)	34.6 (5.5)	35.3 (5.1)	5.2 (3.3) **	−0.8	0.580	−0.204	8.0 (4.7) **	0.2	0.887	0.053	8.7 (4.8) **	0.6	0.703	0.141
	CG	33.5 (5.6)	35.3 (5.7)	35.6 (5.4)	6.0 (4.2) **				7.7 (4.2) **				8.0 (4.2) **			
Extension	IG	38.0 (5.3)	37.4 (5.6)	37.9 (5.7)	3.5 (3.6) **	−0.3	0.838	−0.075	2.9 (2.8) **	−1.9	0.084	−0.654	3.4 (3.1) **	−2.0	0.097	−0.628
	CG	32.6 (3.0)	33.6 (4.2)	34.2 (4.3)	3.8 (3.0) **				4.8 (3.0) **				5.4 (3.3) **			
Right Side Bending	IG	43.7 (2.2)	43.5 (5.3)	43.5 (2.5)	3.9 (4.5) **	0.5	0.750	0.118	3.7 (4.8) **	−0.5	0.755	−0.115	3.7 (4.7) *	−0.2	0.889	−0.051
	CG	42.0 (2.5)	42.7 (2.7)	42.4 (3.2)	3.5 (3.4) **				4.2 (3.2) **				3.9 (2.9) **			
Left Side Bending	IG	42.8 (2.9)	43.2 (2.1)	43.0 (2.4)	3.9 (3.9) **	0.1	0.956	0.020	4.3 (3.6) **	0.1	0.958	0.019	4.1 (3.4) **	0.0	1.000	0.000
	CG	41.5 (2.9)	41.9 (2.6)	41.73(3.0)	3.9 (2.6) **				4.3 (3.3) **				4.1 (3.3) **			
Right Rotation	IG	50.3 (8.2)	58.7 (6.3)	60.3 (6.3)	7.9 (8.5) **	−0.6	0.824	−0.082	16.3 (8.2) **	5.5	0.053	0.737	17.9 (7.4) **	7.1	0.007 *	1.062
	CG	53.5 (6.2)	55.9 (6.6)	55.9 (6.4)	8.5 (5.9) **				10.8 (6.7) **				10.9 (5.8) **			
Left Rotation	IG	64.0 (7.5)	65.1 (6.2)	65.6 (7.0)	10.6 (8.6) **	5.5	0.031	0.832	11.7 (7.9) **	5.2	0.040	0.788	12.2 (9.2) **	5.6	0.041	0.781
	CG	58.6 (5.6)	60.1 (5.7)	60.1 (5.7)	5.1 (3.8) **				6.5 (5.0) **				6.6 (4.2) **			
Pressure Pain Threshold (kg/cm^2^)																
Right Trapezius	IG	2.8 (0.8)	2.9 (0.7)	3.0 (0.7)	0.8 (0.4) **	0.5	0.010 *	1.015	1.0 (0.4) **	0.5	0.007 *	1.065	1.1 (0.4) **	0.6	<0.001 *	1.386
	CG	2.2 (0.6)	2.4 (0.5)	2.4 (0.5)	0.3 (0.6)				0.5 (0.5) **				0.5 (0.5) **			
Left Trapezius	IG	2.7 (0.8)	2.9 (0.8)	2.9 (0.7)	0.8 (0.4) **	0.4	0.005 *	1.125	1.0 (0.4) **	0.4	0.015 *	0.943	1.1 (0.3) **	0.5	0.004 *	1.141
	CG	2.2 (0.5)	2.4 (0.5)	2.4 (0.4)	0.4 (0.4) **				0.6 (0.5) **				0.6 (0.5) **			
Right Splenius	IG	2.3 (0.6)	2.4 (0.5)	2.5 (0.6)	0.8 (0.3) **	0.3	0.010 *	1.006	0.9 (0.3) **	0.4	0.004 *	1.158	0.9 (0.3) **	0.3	0.006 *	1.098
	CG	1.8 (0.4)	1.9 (0.5)	1.9 (0.5)	0.4 (0.3) **				0.5 (0.4) **				0.6 (0.3) **			
Left Splenius	IG	2.2 (0.7)	2.4 (0.5)	2.4 (0.6)	0.6 (0.3) **	0.3	0.017 *	0.927	0.8 (0.2) **	0.4	0.002 *	1.226	0.9 (0.3) **	0.3	0.007 *	1.058
	CG	1.7 (0.4)	1.8 (0.5)	2.0 (0.4)	0.3 (0.3) **				0.4 (0.4) **				0.6 (0.3) **			

Abbreviations. T1: Post-Treatment; T2: One-month Follow-up; T3: Three-month Follow-up; IG: Intervention Group; CG: Control Group; *p*: *p*-value; d: Cohen’s d; SD: Standard Deviation; MD: Mean Difference; NPRS: Numerical Pain Rating Scale; NDI: Neck Disability Index. * *p* < 0.017 after Bonferroni correction in between-group analysis. ** *p* < 0.0125 after Bonferroni correction in within-group analysis.

## Data Availability

Data are contained within the article.

## Abbreviations

The following abbreviations are used in this manuscript:

NDI	Neck Disability Index
NPRS	Numerical Pain Rating Scale
TENS	Transcutaneous Electrical Nerve Stimulation
tDCS	Transcranial Direct Current Stimulation

## References

[1] Bier J.D., Scholten-Peeters W.G., Staal J.B., Pool J., van Tulder M.W., Beekman E., Knoop J., Meerhoff G., Verhagen A.P. (2018). Clinical Practice Guideline for Physical Therapy Assessment and Treatment in Patients With Nonspecific Neck Pain. Phys. Ther..

[2] Cohen S.P. (2015). Epidemiology, diagnosis, and treatment of neck pain. Mayo Clinic Proceedings.

[3] Cohen S.P., Hooten W.M. (2017). Advances in the diagnosis and management of neck pain. BMJ.

[4] Liu R., Kurihara C., Tsai H.T., Silvestri P.J., Bennett M.I., Pasquina P.F., Cohen S.P. (2017). Classification and treatment of chronic Neck pain: A longitudinal cohort study. Reg. Anesth. Pain Med..

[5] Blanpied P.R., Gross A.R., Elliott J.M., Devaney L.L., Clewley D., Walton D.M., Sparks C., Robertson E.K., Altman R.D., Beattie P. (2017). Clinical practice guidelines linked to the international classification of functioning, disability and health from the orthopaedic section of the American physical therapy association. J. Orthop. Sports Phys. Ther..

[6] Côté P., Yu H., Shearer H.M., Randhawa K., Wong J.J., Mior S., Ameis A., Carroll L.J., Nordin M., Varatharajan S. (2019). Non pharmacological management of persistent headaches associated with neck pain: A clinical practice guideline from the Ontario protocol for traffic injury management (OPTIMa) collaboration. Eur. J. Pain.

[7] Fitzgibbon B.M., Schabrun S.M. (2019). Transcranial Direct Current Stimulation for Pain Disorders: Challenges and New Frontiers. Clin. Pharmacol. Ther..

[8] Andrade Ortega J.A., Martínez A.D.D., Ruiz R.A. (2008). Validación de una versión Española del Índice de Discapacidad Cervical. Med. Clínica.

[9] Malfliet A., Coppieters I., Van Wilgen P., Kregel J., De Pauw R., Dolphens M., Ickmans K. (2017). Brain changes associated with cognitive and emotional factors in chronic pain: A systematic review. Eur. J. Pain.

[10] Nitsche M.A., Paulus W. (2011). Transcranial direct current stimulation—Update 2011. Restor. Neurol. Neurosci..

[11] Deus-Yela J., Soler M.D., Pelayo-Vergara R., Vidal-Samsó J. (2017). Estimulación transcraneal por corriente directa en la fibromialgia: Revisión sistemática. Rev. Neurol.

[12] Martelletti P., Jensen R.H., Antal A., Arcioni R., Brighina F., De Tommaso M., Franzini A., Fontaine D., Heiland M., Jürgens T.P. (2013). Neuromodulation of chronic headaches: Position statement from the European Headache Federation. J. Headache Pain.

[13] Pinto C.B., Teixeira Costa B., Duarte D., Fregni F. (2018). Transcranial Direct Current Stimulation as a Therapeutic Tool for Chronic Pain. J. ECT.

[14] Charvet L.E., Shaw M.T., Bikson M., Woods A.J., Knotkova H. (2020). Supervised transcranial direct current stimulation (tDCS) at home: A guide for clinical research and practice. Brain Stimul..

[15] Ahdab R., Mansour A.G., Khazen G., El-Khoury C., Sabbouh T.M., Salem M., Yamak W., Ayache S.S., Riachi N. (2019). Cathodal Transcranial Direct Current Stimulation of the Occipital cortex in Episodic Migraine: A Randomized Sham-Controlled Crossover Study. J. Clin. Med..

[16] Viganò A., D’Elia T.S., Sava S.L., Auvé M., De Pasqua V., Colosimo A., Di Piero V., Schoenen J., Magis D. (2013). Transcranial Direct Current Stimulation (tDCS) of the visual cortex: A proof-of concept study based on interictal electrophysiological abnormalities in migraine. J. Headache Pain.

[17] Przeklasa-Muszyńska A., Kocot-Kępska M., Dobrogowski J., Wiatr M., Mika J. (2017). Transcranial direct current stimulation (tDCS) and its influence on analgesics effectiveness in patients suffering from migraine headache. Pharmacol. Rep..

[18] Antal A., Kriener N., Lang N., Boros K., Paulus W. (2011). Cathodal transcranial direct current stimulation of the visual cortex in the prophylactic treatment of migraine. Cephalalgia.

[19] Auvichayapat P., Janyacharoen T., Rotenberg A., Tiamkao S., Krisanaprakornkit T., Sinawat S., Punjaruk W., Thinkhamrop B., Auvichayapat N. (2012). Migraine Prophylaxis by Anodal Transcranial Direct Current Stimulation, a Randomized, Placebo-Controlled Trial. J. Med. Assoc. Thail..

[20] Caulfield K.A., Badran B.W., DeVries W.H., Summers P.M., Kofmehl E., Li X., Borckardt J.J., Bikson M., George M.S. (2020). Transcranial electrical stimulation motor threshold can estimate individualized tDCS dosage from reverse-calculation electric-field modeling. Brain Stimul..

[21] DaSilva A.F., Truong D.Q., DosSantos M.F., Toback R.L., Datta A., Bikson M. (2015). State of-art neuroanatomical target analysis of high-definition and conventional tDCS montages used for migraine and pain control. Front. Neuroanat..

[22] DaSilva A.F., Mendonca M.E., Zaghi S., Lopes M., Dossantos M.F., Spierings E.L., Bajwa Z., Datta A., Bikson M., Fregni F. (2012). TDCS-induced analgesia and electrical fields in pain-related neural networks in chronic migraine. Headache J. Head Face Pain.

[23] Woods A.J., Antal A., Bikson M., Boggio P.S., Brunoni A.R., Celnik P., Cohen L.G., Fregni F., Herrmann C.S., Kappenman E.S. (2016). A technical guide to tDCS, and related non-invasive brain stimulation tools. Clin. Neurophysiol..

[24] Ayache S.S., Chalah M.A. (2020). Transcranial Direct Current Stimulation and Migraine The Beginning of a Long Journey. J. Clin. Med..

[25] Andrade S.M., de Brito Aranha R.E.L., de Oliveira E.A., de Mendonça C.T.P.L., Martins W.K.N., Alves N.T., Fernández-Calvo B. (2017). Transcranial direct current stimulation over the primary motor vs prefrontal cortex in refractory chronic migraine: A pilot randomized controlled trial. J. Neurol. Sci..

[26] Straudi S., Buja S., Baroni A., Pavarelli C., Pranovi G., Fregni F., Basaglia N. (2018). The effects of transcranial direct current stimulation (tDCS) combined with group exercise treatment in subjects with chronic low back pain: A pilot randomized control trial. Clin. Rehabil..

[27] Teoli D., Dua A., An J. (2024). Transcutaneous Electrical Nerve Stimulation (TENS). StatPearls.

[28] Martimbianco A.L.C., Porfírio G.J.M., Pacheco R.L., Torloni M.R., Riera R. (2019). Transcutaneous electrical nerve stimulation (TENS) for chronic neck pain. Cochrane Database Syst. Rev..

[29] Qing W., Shi X., Zhang Q., Peng L., He C., Wei Q. (2021). Effect of Therapeutic Ultrasound for Neck Pain: A Systematic Review and Meta-Analysis. Arch. Phys. Med. Rehabil..

[30] de Zoete R.M.J. (2023). Exercise Therapy for Chronic Neck Pain: Tailoring Person-Centred Approaches within Contemporary Management. J. Clin. Med..

[31] Teichert F., Karner V., Döding R., Saueressig T., Owen P.J., Belavy D.L. (2023). Effectiveness of Exercise Interventions for Preventing Neck Pain: A Systematic Review With Meta-analysis of Randomized Controlled Trials. J. Orthop. Sports Phys. Ther..

[32] Binder A. (2007). The diagnosis and treatment of nonspecific neck pain and whiplash. Eura Medicophys..

[33] American Society of Anesthesiologists Task Force on Chronic Pain Management, American Society of Regional Anesthesia and Pain Medicine (2010). Practice Guidelines for Chronic Pain Management: An Updated Report by the American Society of Anesthesiologists Task Force on Chronic Pain Management and the American Society of Regional Anesthesia and Pain Medicine. Anesthesiology.

[34] Prablek M., Gadot R., Xu D.S., Ropper A.E. (2023). Neck Pain: Differential Diagnosis and Management. Neurol. Clin..

[35] Lauche R., Cramer H., Hohmann C., Choi K.E., Rampp T., Saha F.J., Musial F., Langhorst J., Dobos G. (2012). The effect of traditional cupping on pain and mechanical thresholds in patients with chronic nonspecific neck pain: A randomised controlled pilot study. Altern. Med..

[36] Thong I.S.K., Jensen M.P., Miró J., Tan G. (2018). The validity of pain intensity measures: What do the NRS, VAS, VRS, and FPS-R measure?. Scand. J. Pain.

[37] Kinser A.M., Sands W.A., Stone M.H. (2009). Realiability and Validity of a Pressure Algometer. J. Strength Cond. Res..

[38] Lozano A., Morales M., Lorenzo C., Sánchez A. (2006). Dolor y estrés en fisioterapia: Algometría de presión. Rev. Iberoam. Fisioter. Y Kinesiol..

[39] Mäkelä S., Pöntinen P. (1988). Reliability of pressure threshold meter in location of latent trigger points in healthy subjects. Scand. J. Acupunct. Electrother..

[40] Chesterton L., Barlas P., Foster N., Baxter G., Wright C. (2003). Gender differences in pressure pain threshold in healthy humans. Pain.

[41] Maquet D., Croisier J., Demoulin C., Crielaard J. (2004). Pressure pain thresholds of tender point sites in patients with fibromyalgia and in healthy controls. Eur. J. Pain.

[42] Huang T., Zhang C., Han Z., Zhong W., Zhao Z., Zhu Y., Luo X., Zhang J. (2023). A novel rapid measurement method of cervical sagittal parameters based on the integrated inclinometer of a smartphone: A validity and reliability study. Ann. Med..

[43] (2023). IBM SPSS Statistics. https://www.ibm.com/products/spss-statistics.

[44] Cohen J. (1992). A power primer. Psychol. Bull..

[45] Fregni F., El-Hagrassy M.M., Pacheco-Barrios K., Carvalho S., Leite J., Simis M., Brunelin J., Nakamura-Palacios E.M., Marangolo P., Venkatasubramanian G. (2021). Evidence-Based Guidelines and Secondary Meta-Analysis for the Use of Transcranial Direct Current Stimulation in Neurological and Psychiatric Disorders. Int. J. Neuropsychopharmacol..

[46] Lim M., Kim D.J., Nascimento T.D., DaSilva A.F. (2024). High-definition tDCS over primary motor cortex modulates brain signal variability and functional connectivity in episodic migraine. Clin. Neurophysiol..

[47] DaSilva A.F., Kim D.J., Lim M., Nascimento T.D., Scott P.J.H., Smith Y.R., A Koeppe R., Zubieta J.-K., Kaciroti N. (2023). Effect of High-Definition Transcranial Direct Current Stimulation on Headache Severity and Central micro-Opioid Receptor Availability in Episodic Migraine. J. Pain Res..

[48] Kim D.J., Nascimento T.D., Lim M., Danciu T., Zubieta J.K., Scott P.J.H., Koeppe R., Kaciroti N., DaSilva A.F. (2023). Exploring HD-tDCS Effect on mu-opioid Receptor and Pain Sensitivity in Temporomandibular Disorder: A Pilot Randomized Clinical Trial Study. J. Pain..

[49] Alipour A., Mohammadi R. (2024). Evaluation of the separate and combined effects of anodal tDCS over the M1 and F3 regions on pain relief in patients with type-2 diabetes suffering from neuropathic pain. Neurosci. Lett..

[50] Caulfield K.A., George M.S. (2018). The Future of Brain Stimulation Treatments. Psychiatr. Clin. N. Am..

[51] Wójcik M., Vlček P., Siatkowski I., Grünerová-Lippertová M. (2025). Effects of a single tDCS with mirror therapy stimulation on hand function in healthy individuals. Front. Hum. Neurosci..

[52] Bonifácio de Assis E.D., Martins W.K.N., de Carvalho C.D., Ferreira C.M., Gomes R., Rodrigues E.T.d.A., Meira U.M., de Holanda L.J., Lindquist A.R., Morya E. (2022). Effects of rTMS and tDCS on neuropathic pain after brachial plexus injury: A randomized placebo-controlled pilot study. Sci. Rep..

[53] Reichenbach S., Jüni P., Hincapié C.A., Schneider C., Meli D., Schürch R., Streit S., Lucas C., Mebes C., Rutjes A. (2022). Effect of transcutaneous electrical nerve stimulation (TENS) on knee pain and physical function in patients with symptomatic knee osteoarthritis: The ETRELKA randomized clinical trial. Osteoarthr. Cartil..

[54] Cruccu G., Garcia-Larrea L., Hansson P., Keindl M., Lefaucheur J.P., Paulus W., Taylor R., Tronnier V., Truini A., Attal N. (2016). EAN guidelines on central neurostimulation therapy in chronic pain conditions. Eur. J. Neurol..

[55] Garcia-Larrea L., Perchet C., Hagiwara K., André-Obadia N. (2019). At-Home Cortical Stimulation for Neuropathic Pain: A Feasibility Study with Initial Clinical Results. Neurotherapeutics.

